# Supplementary material for: A Global Screen for Assembly State Changes of the Mitotic Proteome by SEC-SWATH-MS
Source: Cell Syst. 2020 Feb 26;10(2):133–155.e6. doi: 10.1016/j.cels.2020.01.001 (PMC7042714; doi:10.1016/j.cels.2020.01.001)

Q6UX04 | CWC27_HUMAN | CWC27 SDCCAG10 UNQ438/PRO871
Monomer MW [kDa]: 53.847 Monomer expected elution fraction: 46

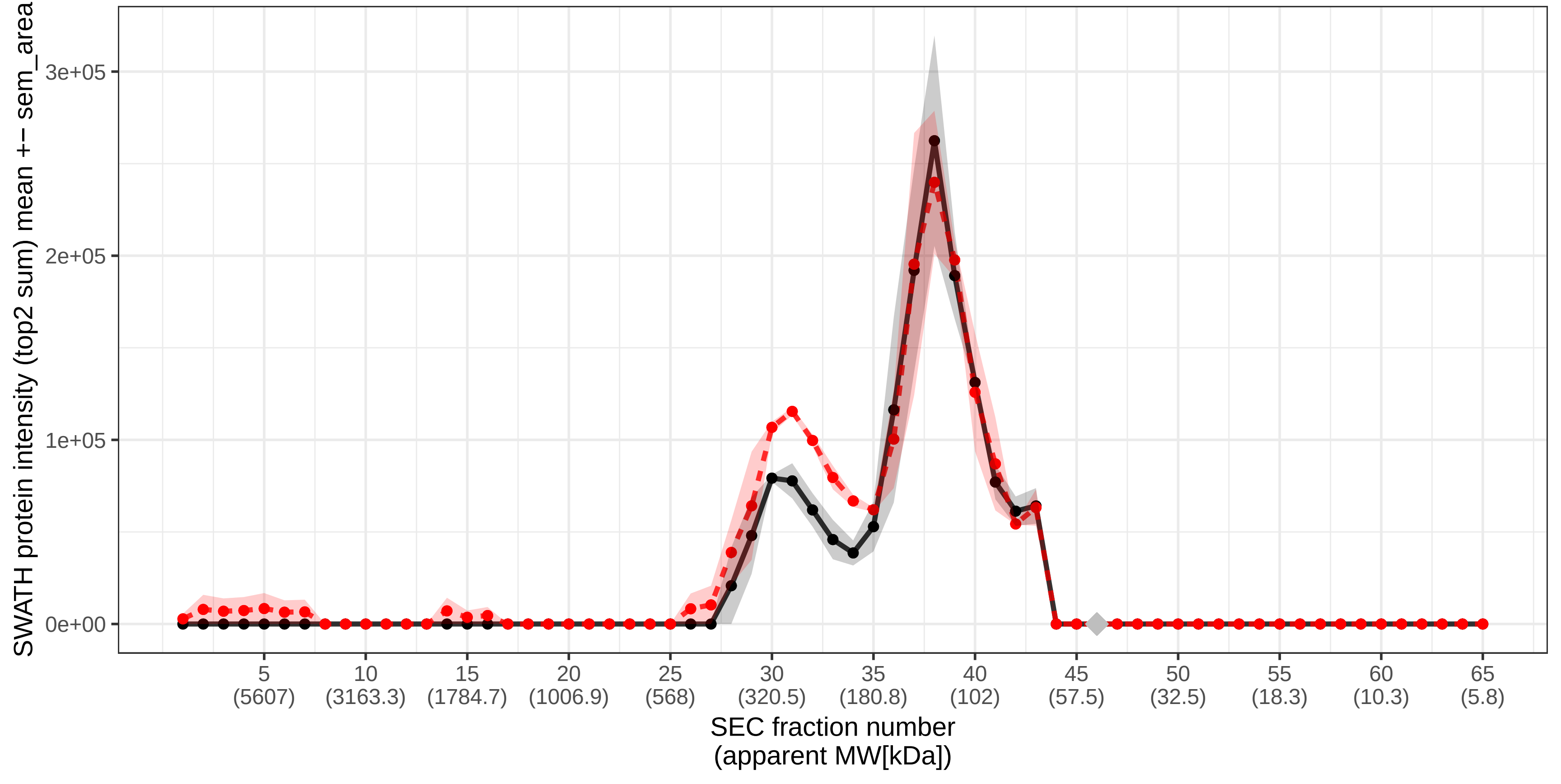

Supplement: Data S1. SEC-SWATH-MS Protein Chromatograms, Related to Figure 1 [file mmc6.zip › SECchrom_Q6UX04_CWC27_HUMAN_CWC27_SDCCAG10_UNQ438_PRO871.pdf]
